# Recovery From Alopecia After COVID-19

**DOI:** 10.7759/cureus.21160

**Published:** 2022-01-12

**Authors:** Yuki Otsuka, Yasuhiro Nakano, Hideharu Hagiya, Kazuki Tokumasu, Fumio Otsuka

**Affiliations:** 1 Department of General Medicine, Okayama University Graduate School of Medicine, Dentistry and Pharmaceutical Sciences, Okayama, JPN

**Keywords:** alopecia areata, covid-19 sequalae, alopecia, covid-19, telogen effluvium

## Abstract

Herein, we report a remarkable case of post-coronavirus disease 2019 (COVID-19) diffuse alopecia that gradually improved and recovered, and the hair volume returned approximately to the pre-infection level, seven months after the patient’s first diagnosis of COVID-19. Approximately 20% of patients with COVID-19 develop alopecia a few months after the acute infection phase. Telogen effluvium is the major type of COVID-19 sequela secondary to physical or psychological distress. It is reversible and is expected to improve without any treatment, and it can be addressed by explaining to the patients their conditions, sharing medical information, and eliminating psychophysical stress by managing systemic complications.

## Introduction

Since November 2021, almost two years have passed since the beginning of the novel coronavirus disease 2019 (COVID-19) pandemic. Studies have revealed that more than one-third of patients with COVID-19 develop a range of persistent symptoms after the acute phase of the infection [1-2]. Some symptoms remain and persist throughout the acute and chronic stages, whereas other symptoms newly appear after the acute phase [3-4]. Hair loss is not common in the acute phase of COVID-19 but is a well-known sequela of COVID-19 observed in approximately 20% of these patients [2]. However, only a few case reports have described its clinical course. Herein, we report the case of a patient with post-COVID-19 alopecia that improved and recovered almost to the pre-infection level.

## Case presentation

**Figure 1:**
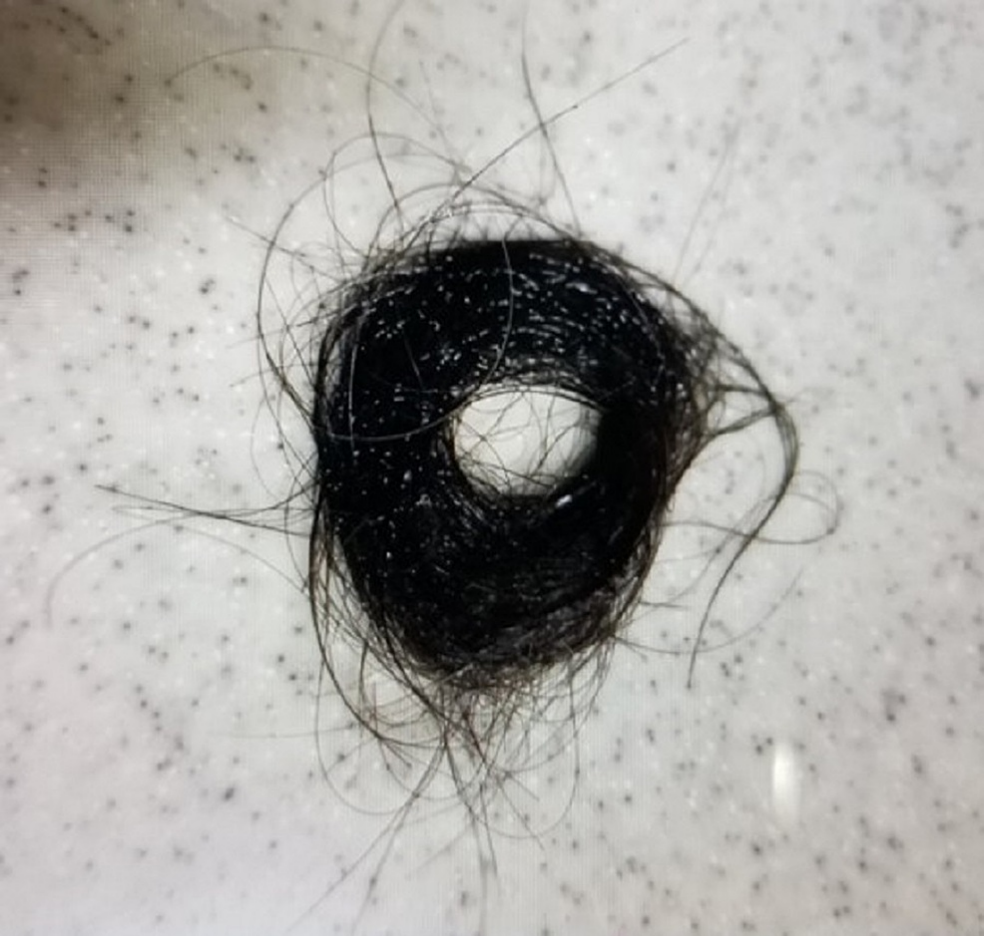
Fallen hair was clogging the shower sink.

A 64-year-old man was diagnosed with severe COVID-19, requiring mechanical ventilation for 11 days. He was treated with a combination of remdesivir, dexamethasone, and tocilizumab, and was discharged from the hospital after one month. One month following discharge, he developed noticeable hair loss and observed shower clogging by the fallen hair daily (Figure 1).

**Figure 2:**
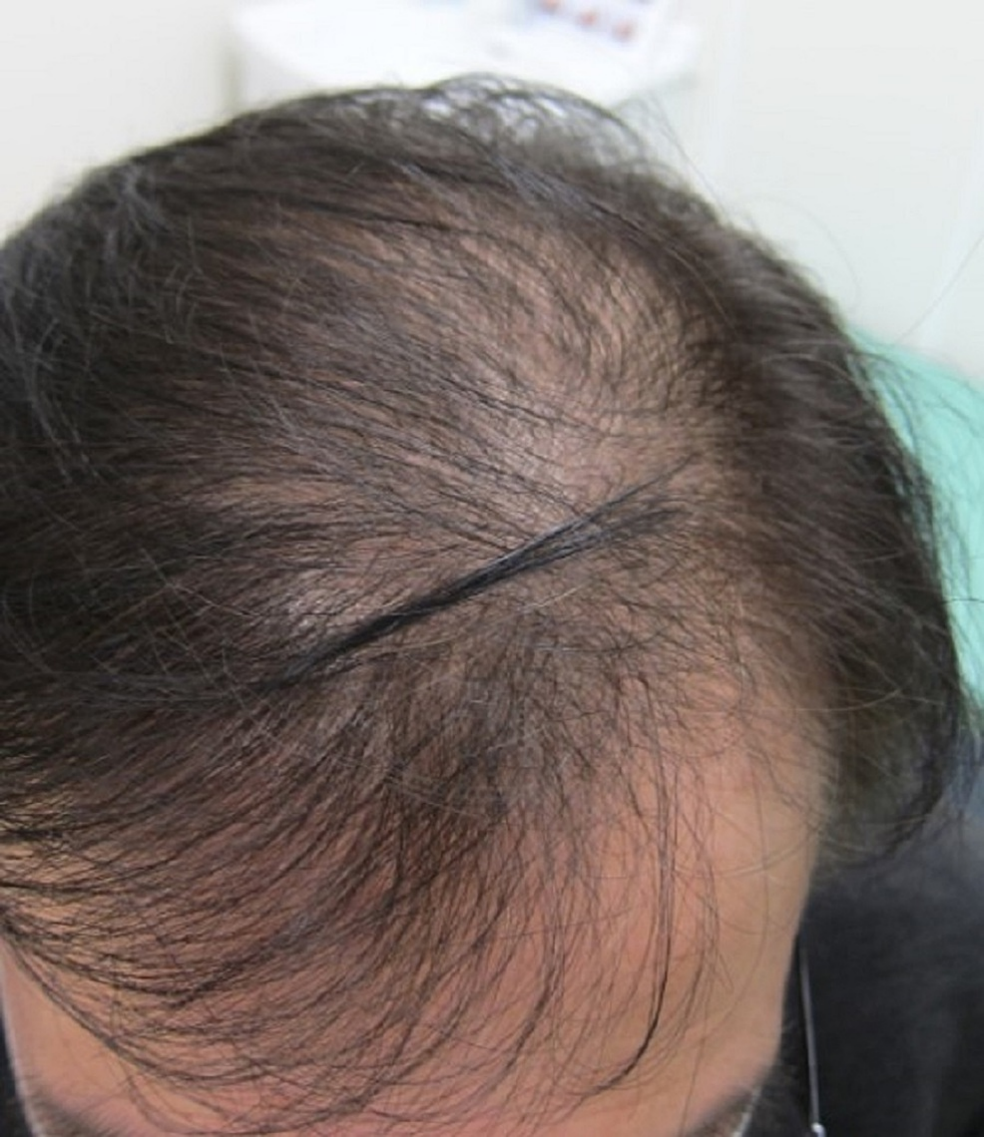
Alopecia gradually spread over the entire head by the first visit.

Because the hair loss continued, the patient visited our COVID-19 aftercare clinic four months after the first onset of COVID-19 [4]. By the first visit, alopecia had spread gradually across the entire head (Figure 2). There were no significant abnormal findings in his laboratory tests other than abnormal serum zinc level or no signs of malnutrition, abnormal thyroid function, or adrenal insufficiency. The serum zinc level was slightly low (76 µg/dL) but not severe to merit supplementation as a deficiency.

**Figure 3:**
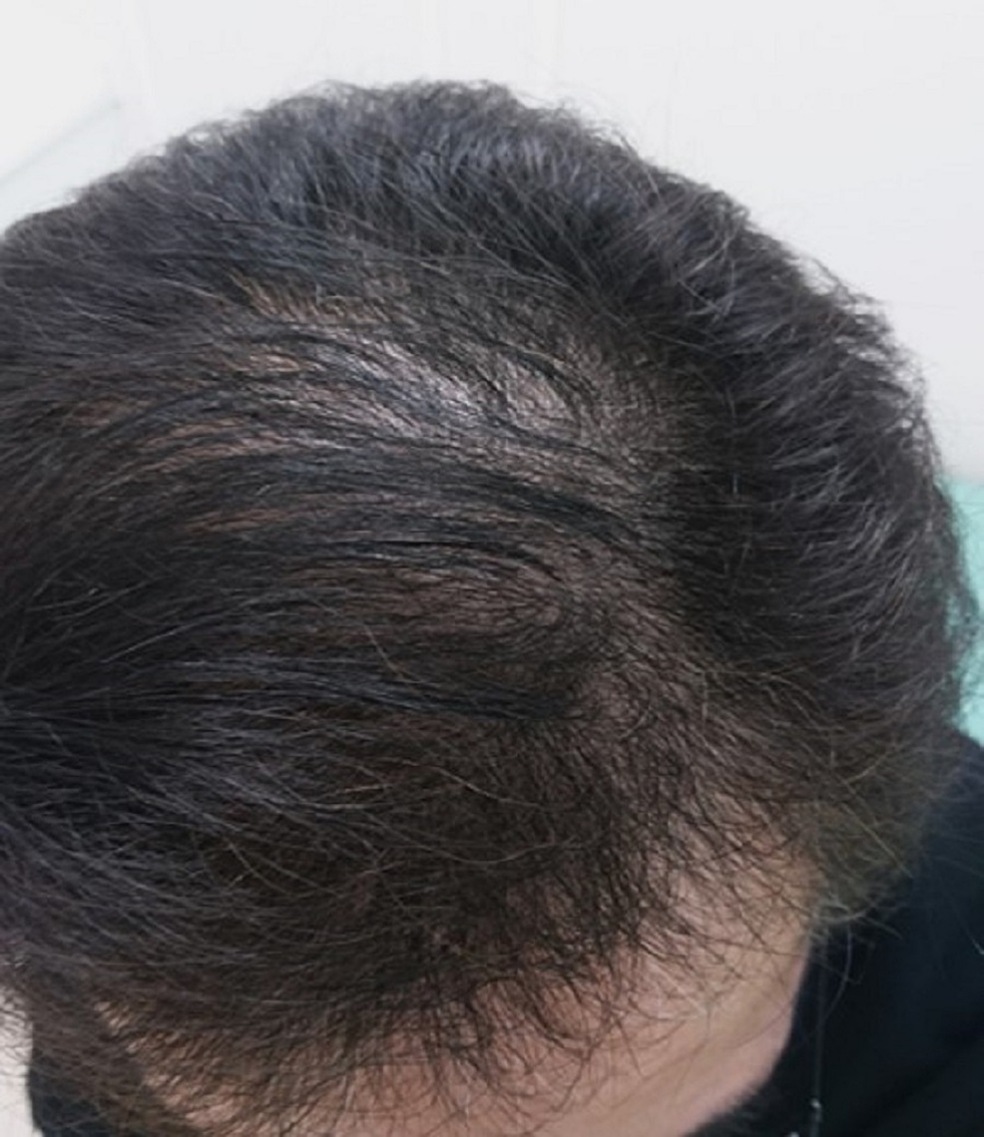
Alopecia gradually recovered seven months after the disease onset.

The alopecia had spread diffusely, and there were no prominent alopecia spots; thus, we suspected the condition to be telogen effluvium (TE). Based on the evidence at that time, no medication was considered necessary, with just observation being sufficient; however, the patient strongly requested a prescription due to anxiety. Therefore, oral cepharanthine (2 mg/day) and topical carpronium were prescribed according to the Japanese guidelines for alopecia areata [5]. His hair loss gradually diminished, and the hair volume recovered to almost the pre-infection level at seven months after the first diagnosis of COVID-19 (Figure 3).

## Discussion

Hair loss is not common in the acute phase of COVID-19, but a sequela of COVID-19 is observed in approximately 20% of the patients [2]. Most cases are TE, secondary to the physical or psychological distress of COVID-19 [6]. Cases of alopecia areata have been reported after COVID-19 [7]. Androgenetic alopecia is considered a risk factor for severe COVID-19 (named the “Gabrin sign”) [8]; however, this is not a sequela.

TE is associated with the telogen phase. Usually, 5%-10% of a person’s hair is in the telogen phase. However, the anagen phase slows down, and more hair is in the catagen and telogen phases in TE. It is observed 3-4 months after physical or psychological distress such as viral infection, emotional stress, severe injuries, surgeries, difficult labor, drugs usage, endocrine disorders, and malnutrition [9]. Even if acute COVID-19 was not severe, the mental stress associated with the infection could cause TE [10].

Although our patient received medication for alopecia areata on his request, TE is reversible and expected to improve without any treatment after the psychophysical damage disappears [6]. The supplementation of iron or zinc can be a treatment option for treating the deficiency of these minerals [9]. Although hair loss is not life-threatening, it can considerably affect the patient’s quality of life; hence, physicians need to provide complete care for such patients [4]. Furthermore, physicians need to reduce anxiety by explaining to patients their conditions, sharing medical information, and eliminating their psychophysical stress by managing various systemic complications. Unfortunately, there are only a few reports, such as our case, wherein the hair loss was recovered to the original state [10]. Thus, we hope that this report will help physicians in encouraging their patients with hair loss after COVID-19.

## Conclusions

Alopecia is a known sequela of COVID-19; most cases are TE secondary to the physical or psychological distress of COVID-19. While patients with alopecia tend to be intensely anxious, the alopecia due to TE is reversible and expected to improve without any treatment. Thus, physicians must encourage the patients by explaining their conditions, sharing medical information, and eliminating their psychophysical stress by managing systemic complications.
